# Migration of Bacteriocins Across Gastrointestinal Epithelial and Vascular Endothelial Cells, as Determined Using *In Vitro* Simulations

**DOI:** 10.1038/s41598-019-47843-9

**Published:** 2019-08-07

**Authors:** Leané Dreyer, Carine Smith, Shelly M. Deane, Leon M. T. Dicks, Anton D. van Staden

**Affiliations:** 1 Department of Microbiology, Stellenbosch University, Stellenbosch, 7600 South Africa; 2 Department of Physiological Sciences, Stellenbosch University, Stellenbosch, 7600 South Africa

**Keywords:** Antimicrobials, Peptides

## Abstract

Little is known about the migration of bacteriocins across human cells. In this study, we report on migration of three bacteriocins nisin, plantaricin 423 and bacST4SA across colonic adenocarcinoma (Caco-2) cells and human umbilical vein endothelial cells (HUVECs). Bacteriocins were fluorescently labelled while still maintaining antimicrobial activity. Migration of fluorescently labelled bacteriocins across monolayers was assessed *in vitro* using transmigration well inserts. After 3 h, 75% of nisin, 85% of plantaricin 423 and 82% of bacST4SA migrated across the Caco-2 cell monolayer. Over the same time span, 88% nisin, 93% plantaricin 423 and 91% bacST4SA migrated across the HUVEC monolayer. The viability of both cell types remained unchanged when exposed to 50 µM of nisin, plantaricin 423 or bacST4SA. The effect of human plasma on bacteriocin activity was also assessed. Activity loss was dependent on bacteriocin type and concentration, with the class-IIa bacteriocins retaining more activity compared to nisin. This is the first report of bacteriocins migrating across simulated gastrointestinal- and vascular-barriers. This study provides some of the first evidence that bacteriocins are capable of crossing the gut-blood-barrier. However, *in vivo* studies need to be performed to confirm these findings and expand on the role of bacteriocin migration across cell barriers.

## Introduction

Several studies have shown the tremendous benefits of probiotics, which are defined as health-promoting bacteria^1–4^. Lactic acid bacteria belonging to the genera *Lactobacillus* and *Bifidobacterium* are most frequently used as probiotics^2,3^. Many of these strains produce antimicrobial compounds, including bacteriocins, which may assist in the control of gastrointestinal infections^5^. Studies using human intestinal epithelial cells and mice have suggested that probiotics may also play a role in maintaining the integrity of the gut-blood barrier (GBB)^6,7^. However, little is known about the migration of bacteriocins across gastrointestinal epithelial and vascular endothelial cells^7^.

Experiments conducted on mice have shown that bacteriocins may have beneficial therapeutic properties *in vivo*. Corr *et al*. (2007) demonstrated that the probiotic strain, *Lactobacillus salivarius* UCC118, produces a bacteriocin (Abp118) *in vivo* that protects mice against infection with *Listeria monocytogenes* EGDe^8^. Nisin F suppressed the growth of *Staphylococcus aureus* in the peritoneal cavity for at least 15 minutes^9^. However, bioluminescence revealed the re-emergence of *S. aureus* after 44 hours, suggesting that nisin F was degraded by proteolytic enzymes or inactivated by non-specific binding^9^. In another study, Brand and co-workers (2011) showed that intraperitoneal administration is an optimal route for treatment with bacteriocins. An immediate decrease in infection was observed after nisin F was injected into mice infected with *L. monocytogenes* EGDe and *S. aureus*^10^. Moreover, nisin F may have a stabilizing effect on the microbiota, since the bacterial population in the gastrointestinal tract (GIT) of mice remained relatively unchanged after nisin F was administered intraperitoneally^11^. Nisin and other bacteriocins have also been shown to have immune modulatory properties^12,13^.

Nisin A, a lantibiotic produced by *Lactococcus lactis* subsp. *lactis*, is active against a number of Gram-positive bacteria, including *Listeria*, *Staphylococcus*, *Bacillus* and *Clostridium* spp.^14–16^. Nisin is one of the most studied bacteriocins, has GRAS (generally regarded as safe) status and is commercially available as Nisaplin®, which is used as a food preservative^17,18^. Nisin binds to lipid II in the bacterial cell wall and prevents cell wall biosynthesis, or forms pores in the cell membrane, leading to depolarization of the cytoplasmic membrane and the efflux of cell components, resulting in cell death^19^. Nisin has also been shown to have immune modulatory properties superior to that of the human cationic peptide LL-37^13^.

The probiotic properties of *Lactobacillus plantarum* 423 and *Enterococcus mundtii* ST4SA have been well studied^20–28^. *Lactobacillus plantarum* 423 adheres to the small intestine of the GIT and *E. mundtii* ST4SA to the lower part of the GIT^24^, preventing gastrointestinal pathogens such as *L. monocytogenes* from attaching to the GIT^20,23,28^. *In vivo* bioluminescence studies conducted on mice^28^ have shown that *L. plantarum* 423 and *E. mundtii* ST4SA, colonise the GIT and prevent *L. monocytogenes* EGDe from causing systemic infections.

Plantaricin 423 (producer: *L. plantarum* 423) is a class IIa bacteriocin, active against a variety of Gram-positive bacteria, including opportunistic pathogens such as *L. monocytogenes* and *Enterococcus faecalis*^19–25,27–29^. BacST4SA (producer: *E. mundtii* ST4SA), also classified as a class IIa bacteriocin, is active against *L. monocytogenes, S. aureus* and *E. faecalis*^21,30^. Both bacteriocins bind to target cells by means of electrostatic interactions between positively charged amino acids and negatively charged phospholipids in the cytoplasmic membrane^29,31^.

Bacteriocins with bactericidal properties, such as those mentioned above, may serve as an alternative to antibiotics and could eradicate a number of bacterial infections, including infections caused by multidrug-resistant strains^32^. Bacteriocins are bactericidal at low (nanomolar) concentrations (*in vitro*) and are not toxic to humans when administered at MIC (minimum inhibitory concentration) levels^7,33,34^. Furthermore, since bacteriocins are ribosomally encoded, genes involved in the production of the mature (active) peptide can be identified and cloned for heterologous production, or the producer strain may be genetically engineered to increase the yield^35,36^.

Drugs administered orally not only need to withstand harsh conditions of the GIT, but also need to cross the highly selective gastro-intestinal epithelial and vascular endothelial cells of the GBB in order to enter the blood stream^7^. Bacteriocins are labile to degradation by proteolytic enzymes in the GIT and are unstable in chemically complex environments such as blood^36,37^. The proteolytic stabilities of therapeutic compounds vary in serum, plasma and fresh blood, because the composition of proteases differs between these blood components^38^. Different proteases recognise different cleavage sites. In blood coagulation, serine proteases are mostly involved, thus cleaving C-terminal lysine or arginine. These proteases also degrade peptides that contain basic residues, such as positively charged histidine. Importantly, peptides can also bind to blood cells and plasma proteins^39^. Therefore, for bacteriocins to be a vaible treatment option they would need to be encapsulated in protective nanoparticles to overcome *in vivo* degradation. Another option is to use probiotic bacteria (encapsulated if needed) as delivery vehicles to constantly secrete bacteriocins in the GIT^1,25^. With recent developments in nanotechnology^40–42^, and the latest knowledge gained on the expression of genes coding for bacteriocin production and secretion^26,31,35,43–45^, target-specific delivery of bacteriocins could soon become a reality. However, apart from limited reports^46,47^, little research has focused on the pharmacodynamics of bacteriocins and not much is known about the migration of these peptides across gut epithelial or vascular endothelial layers^7^.

Mechanisms associated with barrier selectivity are not fully understood. Molecular size and physiochemical properties of substances may play a crucial role^48^. The hydrophobicity of bacteriocins suggests they may interact with epithelial cells in the GIT, but this could also lead to undesired cytotoxicity. Moreover, because of the small size of bacteriocins, they may have the ability to migrate across barriers paracellularly. Human colonic adenocarcinoma (Caco-2) cells are normally used in drug transport studies, since intestinal absorption may determine the clinical success or failure of a therapeutic agent^49^. Human umbilical vein endothelial cells (HUVEC) are enzymatically removed from human umbilical veins and are commonly used in *in vitro* studies, especially in inflammation studies.

**Figure 1:**
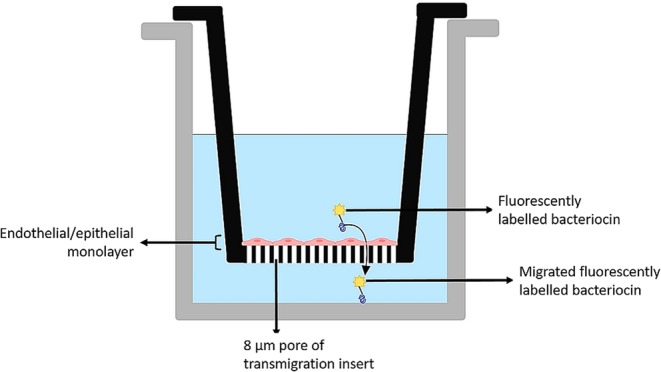
Simulated gastrointestinal epithelial/vascular endothelial barrier constructed with Caco-2 and HUVEC cells, respectively. Transmigration inserts (8 µm pore size) on which the cell lines have been cultured were inserted into wells of a 24-well plate. Figure created in BioRender (https://biorender.io/).

The purpose of this study was thus firstly to produce, purify and characterise selected bacteriocins, and secondly, to assess the capacity of these active bacteriocins to migrate across epithelial/endothelial barriers. We describe the use of Caco-2 and HUVEC monolayers in a Transwell system, to simulate the epithelial and vascular endothelial barriers in the GBB and to study the migration capacity of nisin A, plantaricin 423 and bacST4SA in this context (Fig. 1). We also report on risk for cytotoxic effect of these peptides in the two cell lines. Additionally, stability of the three bacteriocins was monitored in human plasma.

## Results

Bacteriocins nisin A, Plantaricin 423 and BacST4SA were characterised for various properties relevant to their potential for *in vivo* therapeutic effect – these included MIC, stability in plasma and risk of cytotoxicity.

### MIC levels of Nisin A, Plantaricin 423 and BacST4SA

The MIC levels of nisin A, plantaricin 423 and bacST4SA were 8 µM, 12 µM and 10 µM, respectively, as determined against *L. monocytogenes* EGDe.

### Stability in plasma

**Figure 2:**
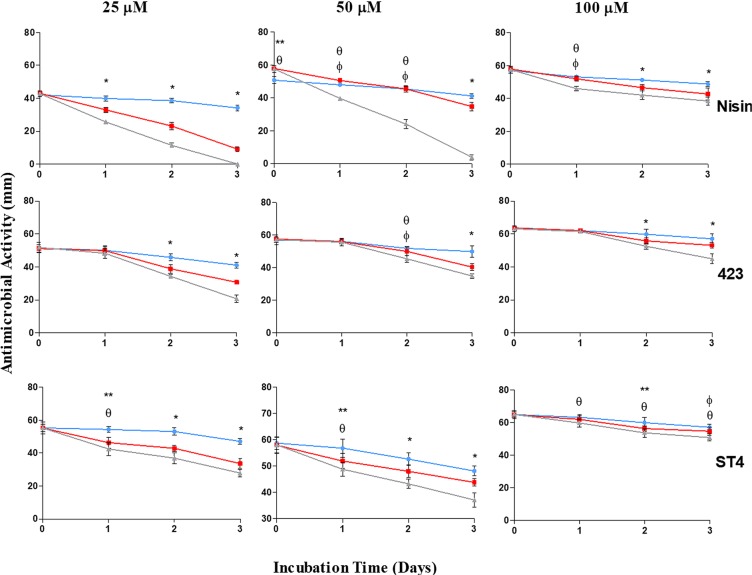
Effect of human blood plasma on the antimicrobial activity of nisin A, plantaricin 423 and bacST4SA over three days. Changes in the antimicrobial activity against *L. monocytogenes* EGDe were observed by spotting the peptides on agar plates and measuring the inhibition zones. Antimicrobial activity of the peptides in the presence of 0% plasma (blue), 40% plasma (red) and 80% plasma (grey) are represented as zone diameter. Statistical differences (p < 0.05) are indicated by symbols above data points (*Differences between all plasma concentrations, **Differences between 0% and 40%, ^θ^differences between 0% and 80%, ^ϕ^Differences between 40% and 80%).

Bacteriocins produced by gut bacteria and able to cross the GBB would enter the circulatory system and as such come into contact with host blood plasma. Thus, in order to have any significant systemic bioactivity and benefit, these peptides would have to be stable in plasma. We therefore investigated bacteriocin stability in terms of antimicrobial activity, when incubated with various concentrations of plasma. After 3 days of incubation in the presence of 40% (v/v) plasma, the antimicrobial activity of 25 µM, 50 µM and 100 µM nisin A decreased by 79%, 40% and 26%, respectively (Fig. 2). After 3 days in the presence of 80% plasma, nisin A (25 µM) lost all of its activity. Higher concentrations of nisin A (50 µM and 100 µM) were slightly more stable in the presence of 80% plasma, as shown by a respective 93% and 33% loss in activity. After 3 days of incubation in the presence of 40% plasma, the antimicrobial activity of 25 µM, 50 µM and 100 µM plantaricin 423 decreased with 40%, 30% and 17%, respectively. In the presence of 80% plasma the activity of 25 µM, 50 µM and 100 µM plantaricin 423 declined with 60%, 39% and 29%, respectively. A similar trend in the decline of activity was recorded for bacST4SA. After 3 days of incubation in the presence of 40% plasma, the antimicrobial activity of 25 µM, 50 µM and 100 µM bacST4SA decreased with 39%, 24% and 16%, respectively. In the presence of 80% plasma an activity decrease of 50%, 36% and 22% was observed for 25 µM, 50 µM and 100 µM bacST4SA, respectively. Statistical differences were observed for various days within the different groups (Fig. 2).

### Cell viability and cytotoxicity of bacteriocins

**Figure 3:**
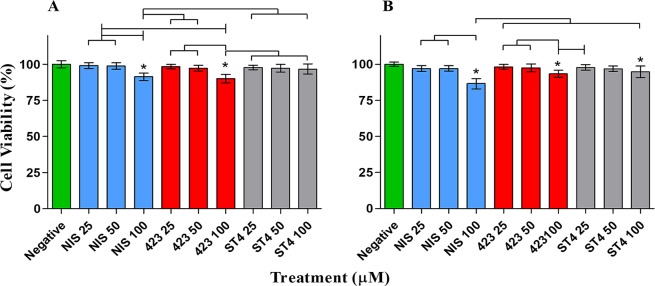
The effect of nisin A (blue), plantaricin 423 (red) and bacST4SA (grey) on cell viability, as recorded using the XTT assay. (**A**) Cell viability of Caco-2. (**B**) Cell viability of HUVECs. Viability after treatment with 25 µM, 50 µM and 100 µM of the peptides are represented as a percentage of total cell viability. Sterile ultrapure water was used as negative control (green) and represents 100% cell viability. Significantly different values are indicated (p < 0.05) above bars with lines and * indicates significant difference with the control.

**Figure 4:**
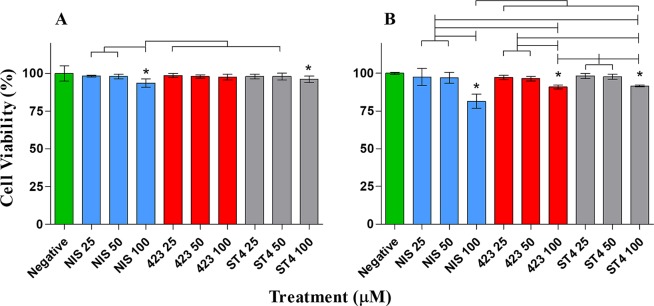
The effect of nisin A (blue), plantaricin 423 (red) and bacST4SA (grey) on cell viability using the neutral red assay. (**A**) Cell viability of Caco-2. (**B**) Cell viability of HUVECs. Viability after treatment with 25 µM, 50 µM and 100 µM of the peptides are represented as a percentage of total cell viability. Sterile ultrapure water was used as negative control (green) and represents 100% cell viability. Significantly different values are indicated (p < 0.05) above bars with lines and * indicates significant difference with the control.

**Figure 5:**
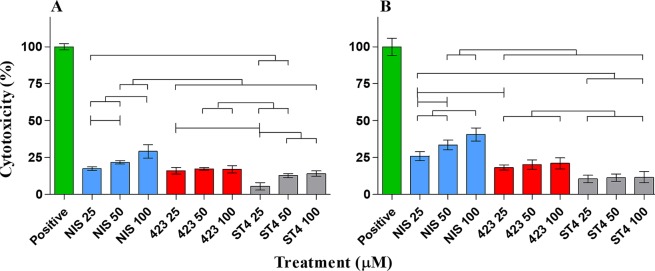
The effect of nisin A (blue), plantaricin 423 (red) and bacST4SA (grey) on plasma membrane integrity using the LDH release assay. (**A**) Cytotoxicity towards Caco-2. (**B**) Cytotoxicity towards HUVECs. Cytotoxicity after treatment with 25 µM, 50 µM and 100 µM of the peptides are represented as a percentage of maximum LDH release. Lysis buffer was used as positive control (green) and represents 100% cytotoxicity. Significantly different values are indicated (p < 0.05) above bars with lines and all treatment groups are significantly differnt to positive control.

In addition to plasma stability, bacteriocins crossing the GBB should not have a detrimental effect on the host, to prevent potentially harmful or undesired effects, such as the instigation of an inflammatory response. In order to investigate the toxicity of bacteriocins to host barrier cells, we evaluated their toxicity against endo- and epithelial cells using three different assays. Results obtained with XTT and neutral red assays showed no significant difference in percentage viability of both cell types after treatment with 25 µM and 50 µM of the three respective bacteriocins (Figs 3 and 4). However, 100 µM nisin A, plantaricin 423 and bacST4SA caused a slight reduction in Caco-2 and HUVEC cell viability. Based on the LDH release from the two cell lines, the relative cytotoxicity of the peptides was significant when compared to the positive control at all three concentrations (Fig. 5). The cytotoxicity levels of 100 µM nisin A, plantaricin 423 and bacST4SA were 41%, 21% and 12% against HUVEC and 29%, 17% and 14% against Caco-2 cells, respectively. Thus, according to the LDH assay nisin A displayed significantly higher levels of cytotoxicity compared to plantaricin 423 and bacST4SA. All three peptides were more toxic to HUVEC than Caco-2 cells, as the overall relative viability of the cells was lower after treatments during the XTT and neutral red assays. In addition, the relative cytotoxicity as assessed by the LDH release assay, increased in a peptide dose-dependent manner. Significant differences were also observed between and within treatment groups (Figs 3–5).

Having established significant antimicrobial action, acceptable plasma stability and a relatively low risk for cytotoxicity, the capacity of the selected bacteriocins to cross gut epithelial and blood vessel endothelial layers was evaluated.

### Peptide labelling

To facilitate effective visualisation and quantification of peptide migration across endo- and epithelial barriers, NHS-fluorescein labelling was used. This technique has previously been shown to have minimal effect on the bioactivity of peptides (namely nisin and vancomycin)^50^. Nisin A, plantaricin 423 and bacST4SA were successfully labelled with NHS-fluorescein, as shown by clear fluorescent zones on agar seeded with *L. monocytogenes* EGDe (not shown). The labelled peptides were, however, less active than the unlabelled peptides, as measured by a decrease in activity (determined by antimicrobial zone sizes) of 32%, 24%, 21% for nisin A, plantaricin 423 and bacST4SA, respectively.

### Migration of bacteriocins across gastrointestinal epithelial cells and vascular endothelial cells

**Figure 6:**
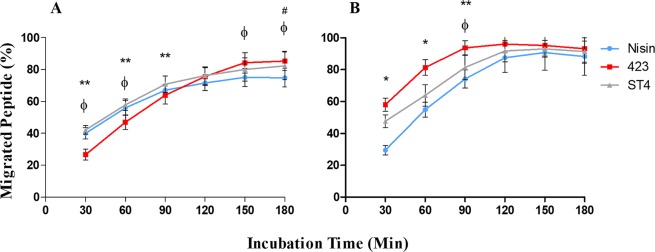
Ability of nisin A, plantaricin 423 and bacST4SA to migrate across a simulated barrier of gastrointestinal epithelial and vascular endothelial cells. (**A**) Migration of peptides across a Caco-2 cell monolayer. (**B**) Migration of peptides across a HUVEC monolayer. Migrated nisin A (blue), plantaricin 423 (red) and bacST4SA (grey) is expressed as percentage of total fluorescence. Statistical differences (p < 0.05) are indicated by symbols above data points. *Differences between all bacteriocins, **Differences between 423 and ST4, ^#^Differences between Nisin and ST4, ^ϕ^Differences between Nisin and 423.

**Figure 7:**
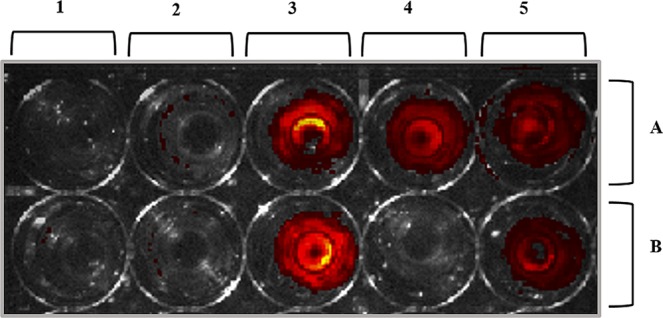
Representative image of fluorescent peptides that attached to or entered the Caco-2 cells (**A**) and HUVECs (**B**) after 3 h of incubation. (1) No peptide or fluorescent marker; (2) NHS-fluorescein; (3) fluorescently labelled nisin A; (4) fluorescently labelled plantaricin 423; (5) fluorescently labelled bacST4SA.

To our knowledge, this is the first study reporting on the ability of bacteriocins to cross the GBB. In order to evaluate migration of bacteriocins across endo- and epithelial cells, a primary 2D monolayer Transwell migration model was used. This model is regularly used in pharmacokinetic studies to assess uptake of pharmaceuticals from the gut^51,52^. Migration of nisin A, plantaricin 423 and bacST4SA across Caco-2 and HUVEC cells is shown in Fig. 6. After 3 h of incubation, 75% of nisin A, 85% of plantaricin 423 and 82% of bacST4SA migrated across the Caco-2 cell monolayer. Migration across HUVEC cells was more rapid, with 88% nisin A, 93% plantaricin 423 and 91% bacST4SA migrating across the monolayer after 3 h. After 3 h similar results were recorded for residual peptide remaining in inserts, compared to the control (no monolayer). The percentage attached and/or entered peptide was calculated; 21% nisin A, 11% plantaricin 423 and 12% bacST4SA remained attached to Caco-2 cells. In the case of HUVECs, 6% nisin A, 0% plantaricin 423 and 3% bacST4SA remained attached or entered the cells (Fig. 7). None of the labelled peptides were cytotoxic to any of the two cell lines, as indicated by trypan blue staining (not shown).

## Discussion

Recently we proposed the possibility of bacteriocins crossing the GBB and hypothesized on their possible role if they do cross^7^. The results in this study provide evidence that bacteriocins are indeed capable, at least *in vitro*, to cross the GBB. Additionally, we also provide data for the bioavailability of these peptides when in contact with human serum. These results provide a framework for future studies to establish the exact mechanism of bacteriocin GBB crossing and justification for future *in vivo* studies.

Since most bacteriocins are membrane active and are hydrophobic, they may attach to cells or other proteins, limiting their availability^10,22^. Furthermore, due to their proteinaceous nature they are also liable to degradation by proteolytic enzymes. In a study by Brand *et al*., 2010, *S. aureus* Xen 36-infected mice were treated with nisin F, which is similar to nisin A used in this study^9^. Nisin F was administered intraperitoneally at a concentration of 640 AU (arbitrary units). Nisin F suppressed the growth of *S. aureus* Xen 36 *in vivo* only for the first 15 minutes. The authors hypothesized that the re-emergence of *S. aureus* was due to degradation of nisin F by proteolytic enzymes. However, as with nisin A, nisin F contains lanthionine bridges which makes it more resistant to proteolytic degradation. In another study nisin F incorporated into bone cement was able to prevent *S. aureus* Xen 36 infection when implanted into an infected subcutaneous pocket^53^. Nisin A and other lantibiotics have also been used to successfully treat and prevent skin infections caused by *S. aureus*^54,55^. Therefore, it is possible that the concentration of nisin used was not high enough to compensate for non-specific binding of nisin to other proteins or membranes. However, peptide removal through immune clearance or proteolytic degradation cannot be discounted without further *in vivo* study. Non-specific binding and/or proteolytic degradation may explain the loss in antimicrobial activity observed for nisin A, plantaricin 423 and bacST4SA after prolonged exposure (1 to 3 days) to blood plasma. Non-specific binding of bacteriocins in the GIT and the GIT environment may also have an effect on the bioactivity of bacteriocins. In the case of antibiotics, delivery to the site of infection is usually within 3 h^33,56^. Chloramphenicol is rapidly absorbed in the GIT when administered orally and reaches peak blood levels within 2 hours^56^. Penicillin G is destroyed by gastric acid and is therefore administered intravenously, whereas penicillin V is more acid-stable with a bioavailability between 60% and 70%. Amoxicillin has an even greater oral bioavailability (74% to 92%). Further *in vivo* experiments are required to determine the optimal dosage of bacteriocins, as high dosages may be toxic.

Previous studies have shown that certain AMPs such as melittin, pediocin and colicin E6 have the ability to interact with mammalian proteins involved in metabolism, disrupt mitochondrial processes, cell structure and cause apoptosis^57,58^. It is therefore crucial to study the interaction of AMPs with mammalian cells to evaluate their potential toxicity before they can be considered for delivery to infectious sites. In the context of the GIT, bacteriocins that are produced by resident- or probiotic-bacteria potentially cross into the circulatory system. Bacteriocin toxicity and interaction with mammalian proteins is therefore equally important as these interactions could result in detrimental/beneficial host responses. Furthermore, the hydrophobicity of nisin A, plantaricin 423 and bacST4SA suggests they may interact with epithelial cells in the GIT and cause undesired toxicity^58^. In this study, nisin A, plantaricin 423 and bacST4SA did not cause significant decreases in HUVEC or Caco-2 cell viability at concentrations up to 50 µM in the XTT and neutral red assays. Results from the LDH release assay indicate that nisin A, plantaricin 423 and bacST4SA have minimal toxicity against Caco-2 cells and HUVECs at concentrations up to 100 µM. These concentrations are, however, well above that required to be effective against sensitive strains *in vitro*. Nisin A, a FDA approved food preservative, displayed significantly higher levels of toxicity compared to plantaricin 423 and bacST4SA in both cell lines. This increase in cytotoxicity could be associated with an increased hydrophobicity, however, the mechanisms involved in the different toxicity levels requires further study. The overall cytotoxicity of nisin A, plantaricin 423 and bacST4SA was greater against HUVECs compared to Caco-2 cells. In a study done by Murinda *et al*. (2003), nisin, pediocin and selected colicins showed different cytotoxicity levels amongst colonic cells and Vero (kidney) cells^57^. These differences may be due to the eukaryotic cell type used. However, other explanations include potential presence of impurities in the AMP preparations used, loss of activity after purification, different exposure times, solvents or media used^58^. However, in the current study the differences in cytotoxicity levels between HUVECs and Caco-2 cells are likely associated with different cell lines used, as the peptides were purified to homogeneity and all solvents were removed. Cytotoxicity studies carried out by Maher (2006) showed that approximately 80 µM nisin A did not have a significant cytotoxic effect on Caco-2 cells^58^. The current study expands on this report by demonstrating that the cytotoxicity of nisin A, plantaricin 423 and bacST4SA indeed only increased at concentrations higher than 50 µM. Even though some toxicity was observed, this was under optimal conditions with elevated bacteriocin concentrations. Factors such as immune inactivation, host clearance mechanisms and other limiting agents, or antagonists, to the bacteriocins were not present. In studies done by van Staden *et al*., (2016) and Heunis *et al*., (2013) lantibiotics were successfully used to treat skin infections without any adverse effects^54,55^. Even though toxicity was not seen in these studies, future *in vivo* studies should be done to evaluate specific toxicity in the GIT and would provide more information regarding the maximum tolerated doses as well as cell type-specific responses to the peptides.

The gastrointestinal barrier is highly selective and prevents the passage of toxic compounds of the luminal microflora whilst allowing the absorption of nutrients from the gut lumen^59^. The molecular size and physiochemical properties of substances play a significant role in this selectivity^48^. Moreover, the vascular endothelial barrier may play a vital role in maintaining the function and structure of the intestinal barrier. Orally administered therapeutic agents need to maintain stability throughout the GIT, cross the intestinal mucous layer, the epithelial barrier and the vascular endothelial barrier to reach the blood stream so that they can be transported to the site of infection. Oral drug delivery vectors such as probiotics can help protect AMPs during GIT transport for effective delivery to infectious sites. Direct oral delivery of nisin A to the site of gastrointestinal colonic infections has also been investigated and is currently under patent^60^. In the current study, a simulation of the gastrointestinal epithelial and vascular endothelial barrier was used to determine if bacteriocins can cross these barriers. Since bacteriocins are extremely small they may diffuse across these barriers by means of the paracellular pathway. Results from this study corroborate this hypothesis. The AMPs, nisin A, plantaricin 423 and bacST4SA can cross a simulation of the epithelial and endothelial layers. Moreover, a small amount of the peptides can attach to or enter epithelial (Caco-2) cells, and nisin A and bacST4SA can attach to or enter endothelial cells (HUVECs). This study did not determine whether the bacteriocins attach to, or enter, the mammalian cells. From our results it is clear that the proportion of bacteriocins that enter/attach cells is not significant compared to the amount that cross the barriers. However, if the bacteriocins entered cells, through mechanisms such as pinocytosis, they did not result in toxicity under the current experimental conditions. Furthermore, the fact that the labelled peptides were not cytotoxic suggests that peptide migration across monolayers did not occur due to pore formation or cell death.

Plantaricin 423 is most effective at migrating across these barriers, but is least effective at attaching to or entering the cells. In contrast, nisin A is the least effective at crossing the barriers but a larger amount of the peptide attached to or entered the cells. Migration across monolayers and association of the peptides with HUVECs or Caco-2 cells differed. Migration across HUVECs was much more rapid with a substantial drop in the association (attachment/entrance) of peptides with HUVECs compared to Caco-2 cells. This could be due to the differences of junctions between the two cell types (i.e. endothelial and epithelial), as well as cell morphology. Additionally, the differences in the ability of the peptides to migrate across the monolayers and associate with cells can be due to differences in hydrophobicity, amino acid composition, or size. However, further investigation is required to elucidate the exact mechanisms involved in bacteriocin-mammalian cell interactions.

In conclusion, current data illustrated that nisin A, plantaricin 423 and bacST4SA have no significant cytotoxicity at effective antimicrobial doses, that they remain stable in blood plasma at these concentrations and that they have the capacity to readily migrate across epithelial and endothelial monolayers. The *in vitro* model used in the current study has limitations and does not fully represent the complex *in vivo* environment of the GIT. Where changes in the structure, function, physiology or pathology of living cells and organs are the focus, organoid models would be preferable. Additionally, advanced visualisation techniques such as microfluidic and confocal microscopy could shed more light on potential occurrence of cell internalisation of peptides, as well as membrane transport mechanisms involved. These results should be followed up by *in vivo* studies, to further elucidate the potential of bacteriocins to cross the GBB under more complex conditions.

## Materials and Methods

SepPak C18 columns were from Waters (Massachusetts, USA). Nisaplin^®^ was from Sigma (Sigma-Aldrich, Missouri, USA). The bicinchoninic acid (BCA) protein assay was from Pierce Biotechnology (Massachusetts, USA). NHS-fluorescein was from Thermo Fisher Scientific (Massachusetts, USA). Cell viability was verified using the neutral red assay (Merck). The XTT (2,3-Bis-(2-Methoxy-4-Nitro-5-Sulphenyl)-2*H*-Tetrazolium-5-Carboxanilide) assay was from Merck. Lactate dehydrogenase (LDH) was determined using the LDH release assay kit (Merck). Human colonic adenocarcinoma (Caco-2) cells, were grown in Dulbecco’s modified Eagle’s medium (DMEM, Merck) and human umbilical vein endothelial cells (HUVEC), in endothelial growth medium (EGM; Lonza, Bail, Switzerland).

### Peptide production and purification

Single colonies of *L. plantarum* 423 and *E. mundtii* ST4SA were inoculated into 10 mL MRS broth, incubated for 18 h at 37 °C and transferred to 2 L MRS broth. After 18 h of incubation at 37 °C, cells were harvested (11 000 × g for 20 min at 4 °C). The pH of the cell-free supernatant was adjusted to 7.0 and heat-treated at 80 °C for 10 min. Bacteriocins were precipitated from the cell-free supernatants with 70% saturated ammonium sulphate, as described previously^61^. Samples were placed on an orbital shaker (200 rpm) at 8 °C for 48 h, the precipitate was collected by centrifugation (20 000 × g, 1 h) and resuspended in sterile phosphate buffered saline (PBS, pH 7.0). The suspension was heat treated at 80 °C for 15 min to bring peptides into suspension and then freeze-dried. The freeze-dried peptides were dissolved in sterile PBS and added to 40 g XAD-16 beads, pre-activated with 80% (v/v) isopropanol containing 0.1% (v/v) TFA. After 24 h on an orbital shaker, the beads were collected and washed with 30% (v/v) ethanol for 15 min. Hydrophobic peptides were eluted from the beads with 70% (v/v) isopropanol containing 0.1% (v/v) TFA. The beads were left in suspension for 18 h on an orbital shaker (100 rpm) at 8 °C. The peptides were separated from the beads by filtering through a 0.45 µM cellulose acetate filter and the isopropanol removed using rotary evaporation at 65 °C (RotaVapor®, Buchi). Peptides were further purified using Sep-Pak C18 reverse phase columns and eluted using 60% (v/v) isopropanol containing 0.1% (v/v) TFA. Isopropanol was removed with rotary evaporation, active fractions were freeze-dried, and antimicrobial activity was tested by using the agar well diffusion assay^54^. Nisaplin^®^ was dissolved in 25% acetonitrile (v/v) containing 0.1% (v/v) TFA and centrifuged to remove undissolved particles. The supernatant was loaded onto a Sep-Pak C18 column and eluted with 40% (v/v) acetonitrile containing 0.1% (v/v) TFA. Active fractions were freeze-dried and tested for activity as mentioned previously.

Plantaricin 423 and bactST4SA were further purified by fast protein liquid chromatography (ÄKTA purifier). Samples were dissolved in 10% (v/v) acetonitrile containing 0.1% (v/v) TFA and added to a HiScale Source 15RPC column (50 × 16 mm, GE Healthcare Life Sciences). Peptides were eluted with an increasing gradient of 10% (v/v) to 55% (v/v) acetonitrile containing 0.1% (v/v) TFA. Active samples were freeze-dried, dissolved in 10% (v/v) acetonitrile and loaded onto a Discovery BIO Wide Pore C18 HPLC (high performance liquid chromatography) column (10 µm, 250 × 10 mm). Plantaricin 423 and bacST4SA were eluted in an acetonitrile gradient of 25% (v/v) to 60% (v/v) acetonitrile and nisin in an increasing gradient of 10% (v/v) to 60% (v/v) acetonitrile containing 0.1% (v/v) TFA. Active peptides were freeze-dried, re-suspended in sterile ultrapure water containing 0.1% (v/v) TFA and analysed by liquid chromatography-mass spectrometry (LC-MS; Central Analytical Facility, Stellenbosch, South Africa).

### Determination of MIC

The BCA assay was used according to the manufactures instructions to determine the concentration of the peptides. The MIC of the peptides was determined using a microtiter plate. Briefly, *L. monocytogenes* EGDe was inoculated into *Listeria* enrichment broth (LEB) and incubated overnight at 37 °C. The culture was inoculated into fresh LEB and incubated until an OD_600_ of 0.1 was reached. A series of peptide concentrations was prepared and 50 µL of each added per well, followed by the addition of 150 µL culture. The controls were ddH_2_O containing 0.1% (v/v) TFA with 150 µL culture or media. Microtiter plates were incubated at 37 °C and OD_600_ readings were taken at T_0_, T_5_ and T_18_ (hours)_._ The MIC was determined as the lowest peptide concentration required to inhibit the growth of *L. monocytogenes* EGDe after 18 h.

### Plasma stability assay

Human blood was collected in tubes with potassium and EDTA as anticoagulant, centrifuged at 500 × g (10 min, 25 °C) and the plasma collected. Different plasma concentrations (0%, 40% and 80%, v/v) were prepared by diluting with sterile ultrapure water. Nisin A, plantaricin 423 and bacST4SA, each suspended in sterile ultrapure water, were added to the plasma to final concentrations of 25 µM, 50 µM and 100 µM, sterile ultrapure water was used as control. Incubation was at 37 °C for 3 days and bacteriocin activity was tested using the agar well diffusion assay. *Listeria monocytogenes* EGDe was used as target organism. Digital images of the plates were taken after incubation and activity quantified by measuring the diameter of the zones using ImageJ software (version 1.5; https://imagej.nih.gov/ij/).

### Cell culture

Caco-2 cells were used between passages 50 and 60 and grown in DMEM. HUVEC were used between passages 5 and 15 and grown in complete EGM. Both cell lines were cultured at 37 °C in a humidified atmosphere in the presence of 5% CO_2_. Cell cultures were routinely sub-cultured before reaching confluence.

For viability and cytotoxicity studies, cells were seeded in 96-well flat-bottom culture plates (1 × 10^4^ cells/well). To determine migration of nisin A, plantaricin 423 and bacST4SA across the gastrointestinal epithelial and vascular endothelial barriers, HUVEC and Caco-2 cells were grown on an 8 µm pore size tissue culture insert, as shown in Fig. 1. All cell culture experiments were done in triplicate and have been repeated more than three times.

### Viability and cytotoxicity assays

#### XTT assay

Cells were grown in 100 µL of culture medium for 24 h at 37 °C. The bacteriocins were separately added to the cells at concentrations of 25 µM, 50 µM and 100 µM. Sterile ultrapure water was used as control. After 24 h of incubation at 37 °C, the reaction mixture containing PMS (N-methyl-dibenzopyrazine-methylsulfate/phenazine methosulphate) and XTT solution, was added to each well and incubated for 4 h at 37 °C. Absorbance readings were recorded at 450 nm, using a microtiter plate reader. The percentage viability was calculated as A_T_/A_C_ × 100; where A_T_ is the absorbance of peptide-treated cells and A_C_ is the absorbance of control (untreated cells).

#### Neutral red assay

Cells were treated with different concentrations of the peptides as described for the XTT assay. Sterile ultrapure water was used as control. After incubation at 37 °C for 24 h, neutral red solution was added to the cells (10% of the culture volume). After 4 h of incubation at 37 °C, the medium was removed by aspiration and the cells were rinsed with neutral red fixative. The fixative was removed, the dye solubilized by adding neutral red and the cells were gently stirred for 10 min on a gyratory shaker. Absorbance readings were recorded at 540 nm and 690 nm, respectively, using a microtiter plate reader. The percentage viability was calculated as the absorbance of treated cells divided by the absorbance of untreated cells (A_T_/A_C_ × 100).

#### LDH release assay

HUVEC and Caco-2 cells were exposed to three different concentrations (25 µM, 50 µM and 100 µM) nisin A, plantaricin 423 and bacST4SA and the presence of extracellular LDH determined using the LDH release assay kit. Sterile ultrapure water was used as negative control (referred to as spontaneous LDH activity) and lysis buffer (10X) was used as positive control (referred to as maximum LDH activity). After incubating the cultures in the presence of the peptides for 24 h at 37 °C, culture supernatants were pipetted into a 96-well plate. Reaction mixture was added to the supernatants and plates were incubated at 25 °C for 30 min. Absorbance was measured at 490 nm and 680 nm, respectively. The percentage cytotoxicity was calculated as follows (1):

1 $$ \% {\rm{Cytotoxicity}}=\frac{({\rm{Compound}}-{\rm{treated}}\,{\rm{LDH}}\,{\rm{activity}})-({\rm{Spontaneous}}\,{\rm{LDH}}\,{\rm{activity}})}{{\rm{Maximum}}\,{\rm{LDH}}\,{\rm{activity}}-{\rm{Spontaneous}}\,{\rm{LDH}}\,{\rm{activity}}}\times 100$$

### Labelling peptides with NHS-fluorescein

HPLC purified stock solutions of nisin A, plantaricin 423 and bacST4SA were prepared by dissolving the peptides in sterile ultrapure water. Immediately before conjugating NHS-fluorescein to the peptides, a stock solution of NHS-fluorescein was prepared by dissolving 1 mg of reagent in 100 µL DMF. The amount of NHS-fluorescein to be added to each peptide was calculated using the Eqs (2) and (3):

2 $${\rm{mL}}\,{\rm{peptide}}\times \frac{{\rm{mg}}\,\mathrm{peptide}\,}{{\rm{mL}}\,{\rm{peptide}}}\,\times \frac{{\rm{mmol}}\,\mathrm{peptide}\,}{{\rm{mg}}\,{\rm{peptide}}}\times \frac{15\,{\rm{mmol}}\,{\rm{NHS}}}{{\rm{mmol}}\,{\rm{peptide}}}={\rm{mmol}}\,{\rm{NHS}}$$

3 $${\rm{mmol}}\,{\rm{NHS}}\times \frac{473.4\,({\rm{MW}}\,{\rm{of}}\,{\rm{NHS}})}{{\rm{mmol}}\,{\rm{NHS}}}\times \frac{100\,\mu L\,\mathrm{DMF}\,}{1\,{\rm{mg}}}=\mu {\rm{L}}\,{\rm{NHS}}$$

The calculated concentration of NHS-fluorescein was added to the peptides and incubated on ice for 2 h. Non-reacted NHS-fluorescein was removed by Sep-Pak C18 column chromatography. Plantaricin 423 and bacST4SA were eluted with 60% acetonitrile containing 0.1% TFA and nisin A with 40% acetonitrile containing 0.1% TFA. Peptides were freeze dried and resuspended in sterile ultrapure water. Concentrations of the labelled peptides were determined with the BCA assay and diluted to 50 µM. Labelled peptides were filtered through a 0.45 µm HVLP membrane and spotted (20 µL) onto LEB agar seeded with 1% *L. monocytogenes* EGDe (OD_600_ 0.1). Plates were incubated overnight at 37 °C and the fluorescent peptides visualised under a UVP 3UV Ultraviolet Lamp. Inhibition zones were measured using ImageJ software and compared to those of non-labelled peptides.

### Migration of bacteriocins across gastrointestinal epithelial cells and vascular endothelial cells

HUVECs and Caco-2 cells were seeded onto transmigration inserts (Fig. 1) at a cell density of 1 × 10^5^ cells/insert (200 µL). Inserts were carefully placed in a 24-well tissue culture plate containing 700 µL cell growth media. Cells were incubated at 37 °C in a humidified atmosphere in the presence of 5% CO_2_, until confluency was reached, and a monolayer formed. The integrity of the monolayers was monitored by ensuring trans-epithelial electrical resistance (TEER) readings remained above 300 Ω throughout the experiment (Millicell-ERS volt-ohm meter, Merck)^51,52,62^. Fluorescently labelled bacteriocins were added to the apical side of the inserts at a concentration of 50 µM, respectively. Transmigration inserts containing no monolayer, was used to determine the total fluorescence. Samples were incubated for 3 h while absorbance was measured at 490 nm every 30 min using a microplate reader. Before each reading, inserts were removed and placed into new microplates containing fresh media. The amount of migrated peptide was calculated as a percentage of total fluorescence in wells (without monolayer) (4):

4 $${\rm{ \% }}\,{\rm{M}}{\rm{i}}{\rm{g}}{\rm{r}}{\rm{a}}{\rm{t}}{\rm{e}}{\rm{d}}\,{\rm{p}}{\rm{e}}{\rm{p}}{\rm{t}}{\rm{i}}{\rm{d}}{\rm{e}}=\frac{{\rm{A}}{\rm{b}}{\rm{s}}{\rm{o}}{\rm{r}}{\rm{b}}{\rm{a}}{\rm{n}}{\rm{c}}{\rm{e}}\,{\rm{i}}{\rm{n}}\,{\rm{w}}{\rm{e}}{\rm{l}}{\rm{l}}\,({\rm{c}}{\rm{o}}{\rm{n}}{\rm{t}}{\rm{a}}{\rm{i}}{\rm{n}}{\rm{i}}{\rm{n}}{\rm{g}}\,{\rm{m}}{\rm{o}}{\rm{n}}{\rm{o}}{\rm{l}}{\rm{a}}{\rm{y}}{\rm{e}}{\rm{r}})}{{\rm{A}}{\rm{b}}{\rm{s}}{\rm{o}}{\rm{r}}{\rm{b}}{\rm{a}}{\rm{n}}{\rm{c}}{\rm{e}}\,{\rm{i}}{\rm{n}}\,{\rm{c}}{\rm{o}}{\rm{n}}{\rm{t}}{\rm{r}}{\rm{o}}{\rm{l}}\,{\rm{w}}{\rm{e}}{\rm{l}}{\rm{l}}\,({\rm{n}}{\rm{o}}\,{\rm{m}}{\rm{o}}{\rm{n}}{\rm{o}}{\rm{l}}{\rm{a}}{\rm{y}}{\rm{e}}{\rm{r}})}\times 100$$

After 3 h, the absorbance of the residual labelled peptide in the inserts (non-migrated peptide) was measured after transferring the non-migrated peptide to 24-well plates containing culture media to a final volume of 700 µl. The percentage residual peptide was calculated as a percentage of total fluorescence (5):

5 $${\rm{ \% }}\,{\rm{R}}{\rm{e}}{\rm{s}}{\rm{i}}{\rm{d}}{\rm{u}}{\rm{a}}{\rm{l}}\,{\rm{n}}{\rm{o}}{\rm{n}}-{\rm{m}}{\rm{i}}{\rm{g}}{\rm{r}}{\rm{a}}{\rm{t}}{\rm{e}}{\rm{d}}\,{\rm{p}}{\rm{e}}{\rm{p}}{\rm{t}}{\rm{i}}{\rm{d}}{\rm{e}}=\frac{{\rm{A}}{\rm{b}}{\rm{s}}{\rm{o}}{\rm{r}}{\rm{b}}{\rm{a}}{\rm{n}}{\rm{c}}{\rm{e}}\,{\rm{i}}{\rm{n}}\,{\rm{i}}{\rm{n}}{\rm{s}}{\rm{e}}{\rm{r}}{\rm{t}}\,}{{\rm{A}}{\rm{b}}{\rm{s}}{\rm{o}}{\rm{r}}{\rm{b}}{\rm{a}}{\rm{n}}{\rm{c}}{\rm{e}}\,{\rm{i}}{\rm{n}}\,{\rm{c}}{\rm{o}}{\rm{n}}{\rm{t}}{\rm{r}}{\rm{o}}{\rm{l}}\,{\rm{w}}{\rm{e}}{\rm{l}}{\rm{l}}\,({\rm{n}}{\rm{o}}\,{\rm{m}}{\rm{o}}{\rm{n}}{\rm{o}}{\rm{l}}{\rm{a}}{\rm{y}}{\rm{e}}{\rm{r}})}\times 100$$

The percentage of peptide that attached to, or entered the cells was quantified by subtracting the sum of migrated peptide (eq. 4) and retained peptide (eq. 5) from the total fluorescence. The cells were washed with PBS and visulized in the IVIS® 100 *In Vivo* Imaging System (Caliper Life Sciences, Hopkinton, US)to detect any labelled peptide that have attached to, or entered, the cells. To determine the cytotoxicity of NHS-fluorescein labelled peptide, a trypan blue exclusion test^63^ was performed. Briefly, cells treated for 3 h with labelled bacteriocins were harvested by centrifugation as described elsewhere and resuspended in 1 ml PBS. One-part cell suspension (100 μl) was added to 100 μl trypan blue (0.4%) and incubated for 3 min at room temperature. The number of unstained (viable) and stained (non-viable) cells was determined using a haemocytometer. The percentage viable cells were calculated by dividing the total number of viable cells per mL with the total number of cells per ml. The integrity of the monolayers was monitored by ensuring TEER readings remained above 300 Ω throughout the experiment.

### Statistical analysis

All data were subjected to analysis of variance (ANOVA) followed by Bonferroni’s Multiple Comparison Test (p < 0.05). Error bars indicate standard deviation. Data was analysed using GraphPad Prism software (version 5.0, USA).

### Ethics statement

Exemption was obtained from the research ethics committee of Stellenbosch University for the purpose of using human donor blood for *in vitro* experiments (reference X15/05/013). All subjects donating blood gave informed consent.

## Data Availability

All data generated or analysed during this study are included in this published article.
